# Toward Physicochemical and Rheological Characterization of Different Injectable Hyaluronic Acid Dermal Fillers Cross-Linked with Polyethylene Glycol Diglycidyl Ether

**DOI:** 10.3390/polym13060948

**Published:** 2021-03-19

**Authors:** Nicola Zerbinati, Sabrina Sommatis, Cristina Maccario, Maria Chiara Capillo, Giulia Grimaldi, Giuseppe Alonci, Marina Protasoni, Raffaele Rauso, Roberto Mocchi

**Affiliations:** 1 Department of Medicine and Surgery, University of Insubria, 21100 Varese, Italy; nicola.zerbinati@uninsubria.it (N.Z.); marina.protasoni@uninsubria.it (M.P.); 2 UB—CARE S.r.l.-Spin-off University of Pavia, 27100 Pavia, Italy; sabrina.sommatis@ub-careitaly.it (S.S.); cristina.maccario@ub-careitaly.it (C.M.); mariachiara.capillo@ub-careitaly.it (M.C.C.); research@ub-careitaly.it (G.G.); 3 Department of Research and Development, Matex Lab Switzerland SA, 1228 Geneve, Switzerland; giuseppe.alonci@neauvia.com; 4 Maxillofacial Surgery Unit, University of Campania “Luigi Vanvitelli”, 81100 Caserta, Italy; dr.raffaele.rauso@gmail.com

**Keywords:** cross-linked HA hydrogels, optical microscopic examination, PEGDE, matrix structure, cohesivity, rheological properties

## Abstract

(1) Background: Injectable hyaluronic acid (HA) dermal fillers are used to restore volume, hydration and skin tone in aesthetic medicine. HA fillers differ from each other due to their cross-linking technologies, with the aim to increase mechanical and biological activities. One of the most recent and promising cross-linkers is polyethylene glycol diglycidyl ether (PEGDE), used by the company Matex Lab S.p.A., (Brindisi, Italy) to create the HA dermal filler PEGDE family. Over the last few years, several studies have been performed to investigate the biocompatibility and biodegradability of these formulations, but little information is available regarding their matrix structure, rheological and physicochemical properties related to their cross-linking technologies, the HA content or the degree of cross-linking. (2) Methods: Seven different injectable HA hydrogels were subjected to optical microscopic examination, cohesivity evaluation and rheological characterization in order to investigate their behavior. (3) Results: The analyzed cross-linked dermal fillers showed a fibrous “spiderweb-like” matrix structure, with each medical device presenting different and peculiar rheological features. Except for HA non cross-linked hydrogel 18 mg/mL, all showed an elastic and cohesive profile. (4) Conclusions: The comparative analysis with other literature works makes a preliminary characterization of these injectable medical devices possible.

## 1. Introduction

Hyaluronic acid (HA) is a naturally nonsulfated glycosaminoglycan that is composed of repeating polymeric disaccharides of D-glucuronic acid and N-acetyl-D-glucosamine linked via a β (1,4)-glycosidic bond [1]. HA is constitutively distributed in several biological tissues and fluids and is involved in important biological functions, such as the regulation of cell adhesion and motility, cell differentiation and proliferation [2]. HA is constitutively found in the adult human body and can be identified in the skin, both in the dermis and the epidermis, where it plays a major role providing constant moisture to the skin through its ability to bind considerable amounts of water. Moreover, HA is associated with collagen and elastin fibers, where it facilitates proper configuration of the extracellular matrix (ECM) networks. With aging, the total amount of HA decreases, contributing to the disorganization of collagen and elastin fibers and to reduced water binding with a consistent loss of moisture. Apparent dehydration, reduced turgidity and loss of elasticity are among the most common changes that characterize skin aging [3]. Over the last few years, increasing attention has been paid to appearance, increasing the demand for HA-based dermal fillers, which have become the material of choice in corrective medical practice. Due to its unique biosafety properties, including full biodegradability, biocompatibility, nontoxicity, rapid bioresorption and nonimmunogenicity, this biopolymer is widely used in soft tissue and dermal correction to create a more youthful appearance [1,4]. In their natural state, HA-based fillers are rapidly metabolized into carbon dioxide and water when injected into normal skin over just a few days. Therefore, to improve their long-lasting stability, chemical or physical modification, such as cross-linking with stabilized agents or hydrophobization, have been developed [5]. 

Chemical cross-linkers are widely used to modify and improve the physical properties of many polymeric materials, conferring a more rigid structure and better-defined shape through chemical linkage [6]. The polymeric networks resulting from linkages between polymer chains can be achieved through various strategies, such as organic, polymerization, quaternization and amidation reactions [7]. The enhanced stability of cross-linkages, either intramolecular or intermolecular, also represents an important advantage for their application as therapeutic cargo [8].

Concerning HA-based fillers, chemical cross-linking is a process that is able to confer a 3D structure to the linear chain of HA through the formation of covalent bonds between HA and a cross-linker agent, improving the biophysical properties while, at the same time, maintaining the biocompatibility and biological activity [9]. Many molecules and polymers are the subject of interest for this purpose; their quality as HA chemical modifiers is determined by the study of two parameters, namely the degree of substitution (DS) and the degree of cross-linking (DC) [10]. The most commonly employed cross-linkers are 1,4-butanediol diglycidyl ether (BDDE), 1,8-diepoxyoctane (DEO), divinyl sulfone (DVS) and polyethylene glycol diglycidyl ether (PEGDE) [4,11]. Cross-linking technologies differ from one manufacturer to another, as does the degree of cross-linking, defined as HA and cross-linker ratio in the final formulation. These differences significantly modify the cohesive and rheological properties of the gels that contribute to the aesthetic outcome. Since the face is subjected to different frequencies and intensities of mechanical stress, understanding the chemical and physicochemical features of fillers may be useful in order to select the ideal product for each corrective application [12]. 

Hydrogels (water-soluble polymers cross-linked via chemical or physical bonds) are a classic example of a viscoelastic material with the storage modulus (G’), which represents the elastic component, exceeding the loss modulus (G’’), the viscous component, when undergoing shear strain. A viscoelastic material presents both elastic and viscous properties; the tangent phase angle (tan δ) can be evaluated to understand which component prevails. The rheological parameter G*, the complex modulus, represents the filler hardness—otherwise, the total energy needed to deform material using shear stress [9,12]. In fact, parameters used to describe how a substance deforms, flows and behaves include viscosity, gel hardness and cohesivity [13]. For cohesivity, as this is a recently explored feature of the HA dermal fillers, no standardized experimental technique has been demonstrated; therefore, scientific opinions on it are mostly controversial [14]. Cohesivity and viscoelastic behavior relate to the ability of fillers to withstand different deformation and forces when implanted in different areas. Fillers with moderate to high G’ can resist shear stress better than those with low G’ [12]. During implantation, gels are subjected to shear stress and vertical compression/elongation forces, both of which cause filler deformation; viscoelastic hydrogels at low stress are gel-like materials, but at increasing shear stress, they can flow, demonstrating a typical shear-thinning behavior. During compression or elongation stress, the shape is retained but the dimensions change [12,13]. 

**Figure 1:**
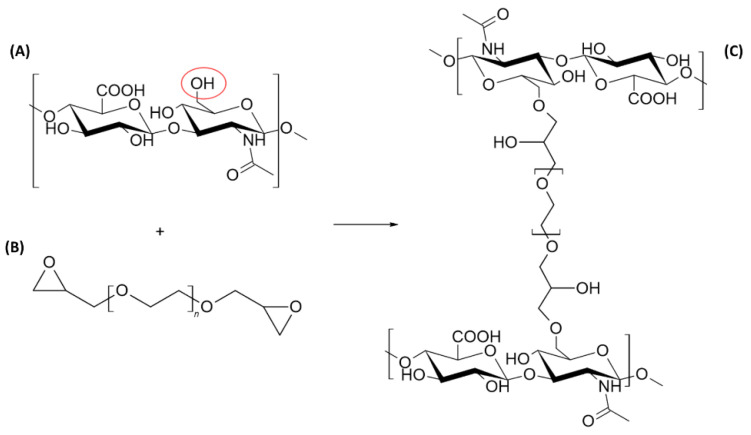
Schematic illustration of cross-linking reaction between hyaluronic acid (HA) (**A**) and the (polyethylene glycol diglycidyl ether) PEGDE cross-linking agent (**B**). The chemical process occurs in alkaline conditions with ether bond formation (**C**). Image obtained with ChemSketch 2020.1.2 (Advanced Chemistry Development, Inc., Toronto, ON, Canada).

This overview presents the rheological and physicochemical properties of different dermal fillers (Matex Lab S.p.A, Brindisi, Italy), through a comparison between seven hydrogels: HA non cross-linked hydrogel 18-mg/mL and 26-mg/mL LR HA have a low cross-linking degree, while the other five gels are more cross-linked but differ in terms of HA concentration (Table 1). Previously, Monticelli et al. performed a chemical characterization of PEGDE-cross-linked dermal fillers, clarifying that the percentage of the cross-linking degree was between 2.8% and 6.2%. The PEGDE concentration is included in the patent property of Matex Lab S.p.a., an Italian company that is part of the medical equipment and supplies manufacturing industry, active in the aesthetic medicine, medical device and wellness equipment market. Their hydrogel production is mainly focused on cross-linking between HA and PEGDE. The organic reaction consists of an epoxide ring opening with the hydroxyl group of the hyaluronic acid (in a basic environment, more nucleophilic than the carboxylic deprotonated group) and the formation of a C–O–C bond, which is among the most stable bonds and consequently is very resistant to degradation (Figure 1) [15,16]. The biocompatibility and biointegration of these fillers were previously assessed [4] but little evidence related to their rheological and physicochemical properties was presented. Therefore, the following study was performed as a preliminary characterization of these gels, correlating matrix structure investigation with rheology and cohesivity properties, in order to provide useful data for a better application of the fillers in aesthetic medical corrections.

## 2. Materials and Methods

### 2.1. Sample Collection

Seven different dermal fillers, provided by Matex Lab S.p.A. (Brindisi, Italy), were investigated regarding their intrinsic matrix organization, their cohesivity and rheological properties. The wide range of fillers is produced through an innovative and advanced SXT (smart cross-linking technology) combining HA and the cross-linker (PEGDE) biopolymer, with a high biosafety and tolerability profile [4]. The quali-quantitative composition of each dermal filler is reported in Table 1, highlighting the differences in hyaluronan concentration and the presence of hydroxyapatite (CaHA).

### 2.2. Sample Preparation and Optical Microscopic Examination

For the microscopic study, the protocol described by Öhrlund and colleagues was taken as a reference [1]. Briefly, 0.1 g of the HA gel was placed into a 9-cm Petri dish containing 10 mL of milli-Q water and 30 µL of Toluidine Blue (Sigma-Aldrich, St. Louis, MO, USA) solution (1% *w*/*v* in water). The Petri dish was placed on a shaker (MS Orbital Shaker, Major Science, Saratoga, CA, USA) for 5 min, allowing the gel particles to dissolve and adsorb the staining. Visualization was performed with an optical inverted microscope (VisiScope, VWR, Radnor, PA, USA) equipped with a digital camera, 5 plus, 5MP (Moticam Camera, Motic, Milan, Italy). Images were taken with 10× magnification and also 4× for the non-cross-linked hydrogel.

### 2.3. Sample Preparation and Cohesivity Evaluation

The cohesivity test was performed according to Sundaram and colleagues’ experimental procedure [2]. The examination was carried out at room temperature. HA hydrogel (1 g) was mixed with 0.1 mg of Toluidine Blue (Sigma-Aldrich, St. Louis, MO, USA) until the dye appeared uniformly distributed within the gel matrix. Later, the gel was extruded into a 1000-mL glass beaker containing 500 mL of milli-Q water and a 2.5-cm magnetic bar stirrer. The height of the syringe from the water’s surface was fixed at 2 cm and the rotational frequency of the magnetic stirrer (VELP Scientifica, Monza, Italy) was set at 160 rpm. Digital images were obtained at the extrusion time (T0), after 15 s (15″), 75 s (75″) and 90 s (90″). Lastly, they were compared with the Gavard–Sundaram Cohesivity Scale, a five-point visual reference scale [2].

### 2.4. Amplitude Sweep Test for Linear Viscoelastic Region (LVER) Determination

Rheological characterization was performed using rotational rheometer Kinexus Plus (Malvern Panalytical, Worcestershire, UK) with a 20-mm plate-plate geometry (PU20 SR2467 SS) and with a working gap set at 1.0 mm. The data processing was performed with rSpace for Kinexus software (Malvern Panalytical, Worcestershire, UK).

The amplitude sweep test was useful to determine the linear viscoelastic region (LVER), where it was possible to work on the samples without damaging their inner structure. In LVER, G’, G’’ and tan δ should be constant at increasing shear strain. The amplitude sweep test allowed determination of the complex modulus (G*), which represents the total energy needed to deform the material under a stress, and the complex viscosity (η*), i.e., the resistance of the gel to flow. The following parameters were set: temperature of 25 °C, shear strain between 0.1 and 1000% and frequency (1 Hz). Subsequently, for further evaluation, temperature (37 °C), shear strain between 0.1 and 10% and frequency (1 Hz) were set.

### 2.5. Evaluation of Dermal Fillers’ Resistance to Elongation

Many articles describe cohesivity as the ability to resist compression/elongation strain [13]. According to this definition, an internal protocol was set up in order to evaluate the cohesivity using the fillers’ resistance to elongation. A known mass of filler (0.4 g) was placed at the working gap and the linear extension test was performed to increase the gap (mm) between the upper and lower geometry at a constant speed. Pictures were taken at selected elongations.

## 3. Results

### 3.1. Optical Microscopic Examination

**Figure 2:**
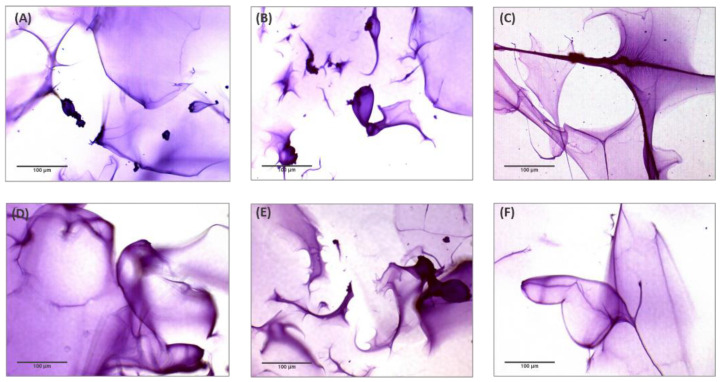
Optical microscopic examination (10×) of six HA dermal fillers cross-linked with PEGDE after staining with Toluidine Blue 1%. (**A**) HA hydrogel 22 mg/mL; (**B**) HA hydrogel 24 mg/mL; (**C)** HA hydrogel 26 mg/mL LR; (**D**) HA hydrogel 26 mg/mL LV; (**E**) HA hydrogel 26 mg/mL with CaHA; (**F**) HA hydrogel 28 mg/mL.

Observation of the tested HA hydrogels by optical microscopy resulted in the visualization of a peculiar matrix structure. Figure 2 shows the images of different microscopic areas of the HA hydrogels obtained with 10× magnification. The investigation demonstrates that the six fillers analyzed, which were obtained by combining HA and PEGDE, did not show remarkable differences in their macrostructure but rather presented a peculiar and homogenous fibrous matrix structure resembling a “spiderweb”.

**Figure 3:**
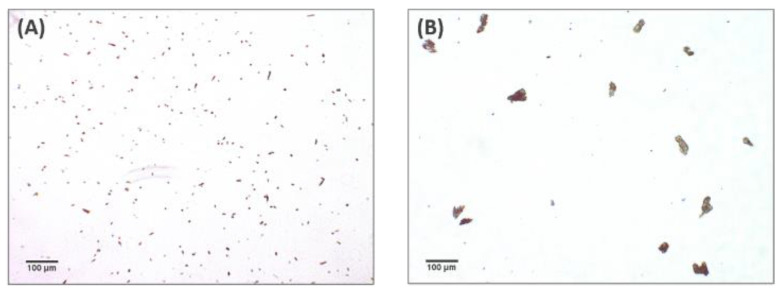
Optical microscopic examination of 18 mg/mL HA non cross-linked dermal filler containing 0.01% CaHA after staining with Toluidine Blue 1%. (**A**) Magnification 4×; (**B**) magnification 10×.

In order to demonstrate that the “spiderweb”-type matrix organization depends on the cross-linker agent, the same analysis was carried out on a non-cross-linked product, containing 18 mg/mL HA and 0.01% CaHA. Figure 3 shows the images obtained with 4× and 10× magnification. The investigation confirmed the absence of a spiderweb-like structure and showed the presence of crystal-like microelements dispersed in the aqueous medium.

### 3.2. Gavard–Sundaram Cohesivity Test

**Figure 4:**
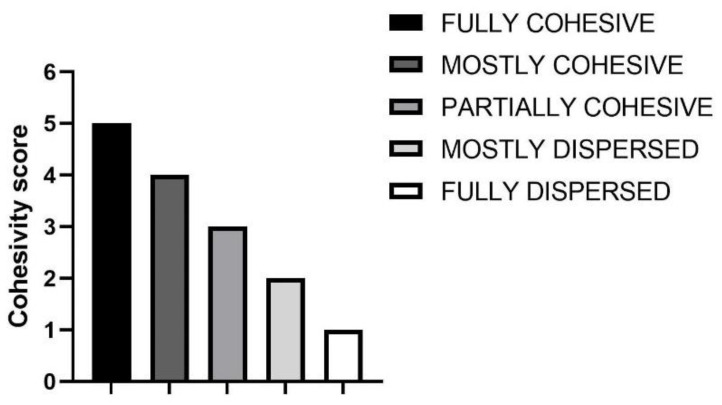
Reproduced Gavard–Sundaram Cohesivity Scale, used as a reference for the data-collected interpretations [2]. Graph obtained with GraphPad Prism 9.0.2 (GraphPad Software, Inc., San Diego, CA, USA).

Cohesivity is a parameter of recent interest, indicating the ability of a gel to withstand stress in physiological conditions [1]. Different methods exist to evaluate cohesivity, with the Gavard–Sundaram Cohesivity Scale being one of the most used. The related scale is shown in Figure 4 [2].

**Figure 5:**
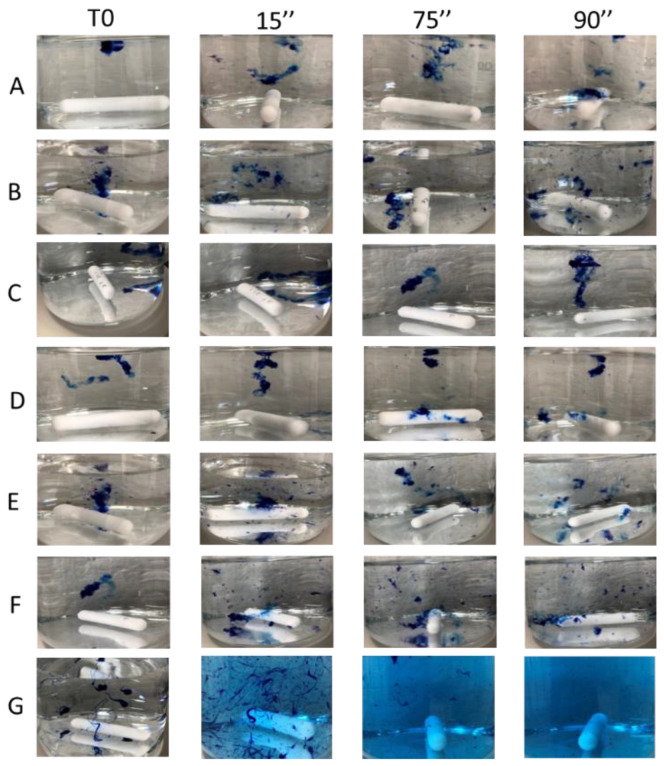
Cohesivity evaluation of six HA dermal fillers cross-linked with PEGDE and a HA hydrogel not cross-linked after staining with Toluidine Blue 1%. (**A**) HA hydrogel 22 mg/mL; (**B**) HA hydrogel 24 mg/mL (**C**) HA hydrogel 26 mg/mL LR; (**D**) HA hydrogel 26 mg/mL LV; (**E**) HA hydrogel 26 mg/mL with CaHA; (**F**) HA hydrogel 28 mg/mL; (**G**) HA non cross-linked hydrogel 18 mg/mL. The images of the gels were captured at extrusion time (T0) and after the conventional times of 15 s (15″), 75 s (75″) and 90 s (90″).

**Figure 6:**
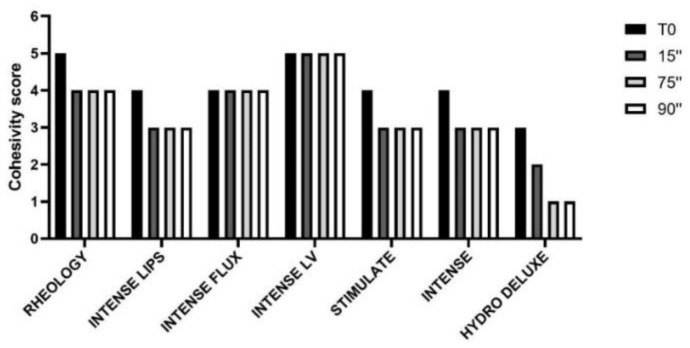
Cohesivity score assigned to six HA dermal fillers cross-linked with PEGDE and a HA non cross-linked dermal filler (18 mg/mL), at different time intervals, according to the Gavard–Sundaram Cohesivity Scale [2]: **1**. fully dispersed; **2**. mostly dispersed; **3**. partially dispersed, partially cohesive; **4**. mostly cohesive; **5**. fully cohesive.

The results obtained from the cohesivity test on the dermal fillers belonging to the PEGDE family and for the non-cross-linked HA hydrogel are reported in Figure 5. The images were captured at the conventional timeframes of 15, 75 and 90 s after starting the test. In Figure 6, the cohesivity scores assigned to the gels according to the Gavard–Sundaram Cohesivity Scale are shown. 

The behavior of the cross-linked HA dermal fillers remained constant throughout all the time ranges analyzed (15″, 75″ and 90″). Instead, the non cross-linked (18 mg/mL HA) filler showed a progressive loss of definition during the shortest timeframes analyzed, with no visible particles detected and the sample appearing as a uniformly colored solution, demonstrating very low cohesivity. Products belonging to the PEGDE family showed a more uniform behavior, demonstrating moderate cohesivity and breaking up into particles partially dispersed into water.

### 3.3. Amplitude Sweep Test

Rheological characterization of seven hyaluronic acid hydrogels was carried out at 25 °C and 37 °C to demonstrate their viscoelastic behavior and the results are shown in Table 2 and Table 3. For each HA hydrogel, the storage modulus (G’), the loss modulus (G’’), the tangent phase angle (tan δ), the complex modulus (G*) and the complex viscosity (η*) parameters were evaluated. All the hydrogels showed that the η* parameter gradually decreased as the shear strain increased outside the linear viscoelastic region (LVER). These seven products demonstrated variable abilities to return to their original shape (“spring back”) and to resist deformation [17]. The term viscosity refers to the capacity to flow from the needle, while G’ refers to the stiffness of the gel and therefore to its ability to resist deformation caused by skin tension and due to facial movements.

### 3.4. Resistance to Elongation

**Figure 7:**
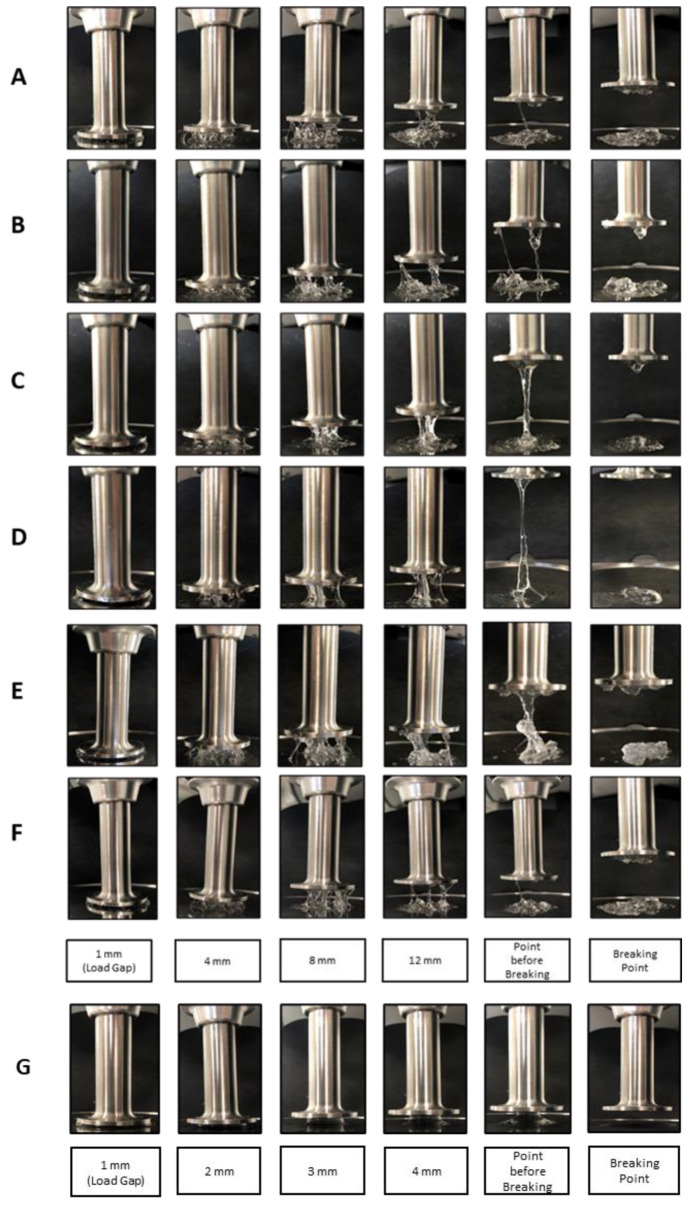
Vertical elongation comparison between seven dermal fillers illustrating the tendency of a cohesive product to stick to itself. (**A**) HA hydrogel 22 mg/mL; (**B**) HA hydrogel 24 mg/mL; (**C**) HA hydrogel 26 mg/mL LV; (**D**) HA hydrogel 26 mg/mL LR; (**E**) HA hydrogel 26 mg/mL with CaHA; (**F**) HA hydrogel 28 mg/mL; (**G**) HA non cross-linked hydrogel 18 mg/mL. For each sample, the load gap (1 mm) and the standard intermediate gaps of 4-8-12 mm and 2-3-4 mm, respectively, for the cross-linked (**A**–**F**) and non-cross linked (**G**) fillers, were evaluated together at the point before breaking and at the breaking point.

Cohesivity can be defined also as the ability to withstand vertical elongation. For this reason, an internal method was developed to evaluate this feature. In Figure 7, the behavior after constant elongation stress of the different dermal fillers is shown. In Table 4, the breaking points for each HA hydrogel, both cross-linked and not, are reported.

## 4. Discussion

Dermal hyaluronic acid fillers are widely used in medical rejuvenation applications to restore lost volume and revitalize skin appearance [3]. Matex Lab S.p.A. focused its research toward an innovative and advanced SXT cross-linking technology based on the use of PEGDE as a cross-linker. PEGDE is a difunctional, highly water-soluble polymer, which is nontoxic and nonimmunogenic; its chemical structure makes the final polymeric organization of the hydrogel less rigid than other cross-linking chemical agents. Moreover, the ether bonds formed during the cross-linking reaction are particularly stable in the physiological conditions of the dermis [4]. Nevertheless, as PEGDE was introduced only recently, an in-depth analysis of the physicochemical and rheological properties is required; in particular, the challenge is to investigate the relationship between matrix structure, rheological properties, cross-linking technologies, HA content and degree of cross-linking to better characterize these hydrogels. Therefore, our study was focused on the investigation of the structural matrix, the cohesive and rheological properties of dermal fillers cross-linked with PEGDE containing different concentrations of hyaluronic acid. A non cross-linked 18 mg/mL hydrogel was selected to compare the physicochemical behavior in the presence or absence of the cross-linker. 

The optical microscopy examination was performed after staining the hydrogels with Toluidine Blue, an acidophilic metachromatic dye that selectively stains acidic tissue components. Toluidine Blue is a member of the thiazine group and is partially soluble in both water and alcohol; in aqueous solution, its amino groups are protonated, leading to the formation of NH_3_^+^ groups that act as cations, showing high affinity toward hyaluronic acid negative charges [5]. Several studies on the matrix structure were previously performed using currently available cross-linked HA gels. Mondon and colleagues [10] demonstrated the possibility of highlighting three HA matrix structures, namely a “spiderweb”-like structure, a particulate structure and an intermediate structure. These differences were detected by optical microscopy, whereas they were less evident with the use of cryoscanning electron microscopy (cryo-SEM) [9]. The investigation demonstrated that the six analyzed fillers cross-linked with PEGDE showed a peculiar and homogenous fibrous matrix structure resembling a “spiderweb”. The same concept could not be demonstrated for the non cross-linked hydrogel (18 mg/mL). 

This behavior shows that non-cross-linked hyaluronic acid has higher solubility than cross-linked fillers, resulting in lower cohesivity and a greater spread ability, as demonstrated by our studies (Figure 5 and Figure 6). All the analyzed cross-linked hydrogels present a fibrous/porous network with different levels of homogeneity; on the basis of the description provided by Mondon [10], this spiderweb organization is a feature closely related to the category of monophasic gels. The correct classification is useful for clinicians to create a natural-looking correction based on the properties of each filler. It was demonstrated that monophasic or biphasic gels interact differently with different areas of the body. 

Cohesivity is an important topic for many manufacturers but there are currently no standardized instruments or methodology acknowledged by the scientific community for its evaluation. Its definition is often influenced by the application field; according to Sundaram, it is defined as the ability of a material not to dissociate in aqueous medium, while in the rheological context, cohesion is defined as the ability to resist compression/elongation stress. For a more complete assessment of cohesivity, we integrated the study of the behavior in water (physiological condition), the rheological data and the evaluation of the resistance to elongation. The protocol proposed by Sundaram represents a qualitative way to investigate this intrinsic property based on the subjective assessment of photographs without the discrimination of large or small particles [18]. Many studies correlated cohesivity with the rheological properties of the filler. In particular, Edsman et al. conducted a study in order to demonstrate a possible correlation between G’ and cohesivity, where gels with higher G’ values showed less cohesivity while fillers with lower G’ values demonstrated higher cohesion values. Commonly, high G’ is an index of better resistance to skin tension [17]. 

In this study, the rheological and physicochemical properties for seven hyaluronic acid fillers were evaluated and the analysis revealed that non cross-linked hydrogel (18 mg/mL) had the lowest values of G’, tan δ, G* and η*. Moreover, it was the only product that showed a G’’ value higher than G’ because the liquid component prevailed over the solid component, as confirmed by the elongation test. Other cross-linked hydrogels, excluding the HA hydrogel 26 mg/mL LR, where a lower concentration of the cross-linker was used [15], showed rheological parameters that increased in direct proportion to the increase in the concentration of hyaluronic acid. HA hydrogel 26 mg/mL LR, with the lowest value of G’ and the highest value of cohesivity in the elongation resistance test, showed a tendency to stick together under stress to recover its shape. The highest values of G’ and G* obtained for HA hydrogel 26 mg/mL with CaHA and HA hydrogel 28 mg/mL indicated that these gels were able to resist deformation to a greater extent. Confirming the results already obtained by Sundaram, the filler containing CaHA was within the group with high viscosity and high elasticity (G’) [14,19]. Nowadays, there are some suggestions on how cohesivity affects the clinical performance of dermal fillers, but controversial opinions are reported in the literature; in some cases, high cohesivity was stated to indicate a better resistance to mechanical degradation and prevention of gel migration [11]. On the other hand, some studies suggested that high cohesivity was correlated with a greater diffusion of the gel depending on the injection plane [2,12,16]. These features represent an important parameter to distinguish cross-linked fillers with different degrees of cohesivity and, consequently, their best aesthetic field of application.

## 5. Conclusions

Our experimental design was focused on the relationship between matrix structure, cohesivity and rheological properties to predict dermal filler behavior. The six analyzed fillers cross-linked with PEGDE showed a peculiar matrix structure resembling a “spiderweb”, a feature closely related to the category of monophasic gels. For a preliminary physicochemical characterization, the rheological and cohesivity properties were investigated. The cohesivity was evaluated as a multiparametric index, which not only depends on the HA content but is also influenced by other factors, such as the cross-linking degree. However, further studies to better characterize these injectable hydrogels are required. Moreover, viscosity and elasticity should be contextualized with physicochemical features and clinical considerations, such as injection techniques and needles. 

## Figures and Tables

**Table 1:** Description of the seven HA hydrogel dermal fillers provided by Matex Lab S.p.A., which were collected to investigate their microscopic structure, rheological and cohesivity properties.

Product	HA Content (mg/mL)	Cross-Linker
HA hydrogel 22 mg/mL	22	PEGDE
HA hydrogel 24 mg/mL	24	PEGDE
HA hydrogel 26 mg/mL LR	26	PEGDE
HA hydrogel 26 mg/mL LV	26	PEGDE
HA hydrogel 26 mg/mL with CaHA ^1^	26	PEGDE
HA hydrogel 28 mg/mL	28	PEGDE
HA non cross-linked hydrogel 18 mg/mL ^2^	18	Not cross-linked

^1^ Containing 1% CaHA. ^2^ Containing 0.01% CaHA.

**Table 2:** Rheological characterization of seven HA hydrogel dermal fillers provided by Matex Lab S.p.A. obtained at a fixed shear strain (1%) and temperature (25 °C) in their linear viscoelastic region (LVER). Data are represented as averages and standard deviations (SDs).

Product	G’ (Pa)	G’’ (Pa)	G* (Pa)	tan δ	η* (Pa s)
HA hydrogel 22 mg/mL	84.05 ± 2.12	27.13 ± 1.14	88.32 ± 2.15	0.32 ± 0.01	14.05 ± 0.34
HA hydrogel 24 mg/mL	82.34 ± 3.72	31.92 ± 1.49	88.32 ± 3.8	0.39 ± 0.02	14.05 ± 0.60
HA hydrogel 26 mg/mL LR	38.90 ± 8.66	27.96 ± 3.90	47.13 ± 9.74	0.73 ± 0.06	7.62 ± 1.48
HA hydrogel 26 mg/mL LV	91.42 ± 4.84	38.86 ± 2.57	99.34 ± 5.44	0.42 ± 0.01	15.81 ± 0.87
HA hydrogel 26 mg/mL with CaHA	164.67 ± 2.94	55.84 ± 5.07	173.93 ± 4.37	0.34 ± 0.03	27.67 ± 0.70
HA hydrogel 28 mg/mL	172.83 ± 3.02	62.63 ± 5.97	183.83 ± 4.72	0.36 ± 0.03	29.26 ± 0.75
HA non cross-linked hydrogel 18 mg/mL	3.80 ± 0.57	13.09 ± 0.54	13.64 ± 0.60	3.50 ± 0.52	2.17 ± 0.09

**Table 3:** Rheological characterization of seven HA hydrogel dermal fillers provided by Matex Lab S.p.A. obtained at a fixed shear strain (1%) and temperature (37 °C) in their linear viscoelastic region (LVER). Data are represented as averages and SDs.

Product	G’ (Pa)	G’’ (Pa)	G* (Pa)	tan δ	η* (Pa s)
HA hydrogel 22 mg/mL	84.80 ± 6.49	27.00 ± 0.26	89.01 ± 6.25	0.32 ± 0.02	14.17 ± 1.00
HA hydrogel 24 mg/mL	83.42 ± 7.88	29.55 ± 3.85	70.51 ± 27.55	0.35 ± 0.02	14.09 ± 1.36
HA hydrogel 26 mg/mL LR	37.31 ± 3.58	22.81 ± 4.72	43.80 ± 5.02	0.61 ± 0.10	6.97 ± 0.8
HA hydrogel 26 mg/mL LV	91.95 ± 3.66	33.56 ± 2.11	97.89 ± 3.61	0.37 ± 0.03	15.58 ± 0.58
HA hydrogel 26 mg/mL with CaHA	161.17 ± 4.68	46.23 ± 6.87	167.73 ± 6.47	0.29 ± 0.03	26.70 ± 1.04
HA hydrogel 28 mg/mL	171.20 ± 10.34	60.34 ± 4.06	181.33 ± 10.96	0.35 ± 0.00	28.86 ± 1.74
HA non cross-linked hydrogel 18 mg/mL	2.06 ± 0.16	9.57 ± 1.24	9.79 ± 1.24	4.64 ± 0.36	1.56 ± 0.20

**Table 4:** Breaking points obtained by the resistance to elongation test of seven HA hydrogel dermal fillers provided by Matex Lab S.p.A. Data are represented as averages and standard deviations (*n* = 1, replicates = 2).

Product	Breaking Point (mm)
HA hydrogel 22 mg/mL	20 ± 0.00
HA hydrogel 24 mg/mL	21 ± 1.41
HA hydrogel 26 mg/mL LR	61 ± 1.41
HA hydrogel 26 mg/mL LV	30 ± 0.00
HA hydrogel 26 mg/mL with CaHA	20 ± 1.41
HA hydrogel 28 mg/mL	16 ± 0.00
HA non cross-linked hydrogel 18 mg/mL	6 ± 0.00

## Data Availability

The data presented in this study are available on request from the corresponding author.
